# Evaluation of Anionic Eco-Friendly Flocculants Prepared from Eucalyptus Pulps with Diverse Lignin Contents for Application in Effluent Treatment

**DOI:** 10.3390/polym13010025

**Published:** 2020-12-23

**Authors:** Kinga Grenda, José A. F. Gamelas, Julien Arnold, Lorenzo Pellizzer, Olivier J. Cayre, Maria G. Rasteiro

**Affiliations:** 1 CIEPQPF–Chemical Process Engineering and Forest Products Research Centre, Department of Chemical Engineering, Faculty of Sciences and Technology, University of Coimbra, Rua Sílvio Lima, 3030-790 Coimbra, Portugal; kingagrenda@hotmail.com (K.G.); jafgas@eq.uc.pt (J.A.F.G.); lorenzo.pellizzer@hotmail.it (L.P.); 2 AQUA+TECH Specialities, Chemin du Chalet-du-Bac 4, Avully, CH-1237 Geneva, Switzerland; julien.arnold@aquatech-water.com; 3 School of Chemical and Process Engineering, University of Leeds, Woodhouse Lane, Leeds LS2 9JT, UK; O.J.Cayre@leeds.ac.uk

**Keywords:** anionic modification of cellulose, decolouration, bio-polyelectrolytes, wastewater treatment, wood wastes valorisation

## Abstract

Modification of cellulosic-rich materials for the production of cellulose-based polyelectrolytes (PELs) can bring several benefits, such as high biodegradability and low or no toxicity, for numerous applications, when compared with the use of traditional, synthetic PELs. Moreover, cellulose-based PELs originating from wood wastes, contribute to the valorisation of such wastes. In this work, *Eucalyptus* pulps with diverse lignin contents, extracted from *Eucalyptus* wood wastes, were anionized by a two–step reaction procedure (periodate oxidation followed by sulfonation). Applying different reaction times (24–144 h) in the sulfonation step allowed for producing a range of cellulose-based anionic PELs with different characteristics. PELs obtained after 24 and 72 h of sulfonation were thoroughly characterized (Fourier transform infrared and ^1^H nuclear magnetic resonance spectroscopies, anionic group content (elemental analysis), zeta potential and hydrodynamic diameter (dynamic light scattering)) and subsequently evaluated as flocculants in decolouration processes of model effluents (Methylene Blue and Crystal Violet) and an industrial effluent from a textile industry. Furthermore, possible flocculation mechanisms induced by the use of the various PELs are discussed. Results are compared with those obtained with a commonly applied, synthetic flocculant (polyacrylamide). It is demonstrated that it was possible to obtain water-soluble lignocellulosic PELs starting from raw materials with different degrees of purity and that those PELs are promising eco-friendly alternative flocculation agents for the decolouration of effluents.

## 1. Introduction

Less than 1% of earth water supply is available for human consumption [1]. Population growth, industrialisation and/or urbanization has led to a decreasing availability of fresh, safe and clean water leading to water availability becoming a global issue. Furthermore, developed countries are also facing direct discharge of harmful, often toxic, industrial or domestic effluents into natural water reservoirs. Among them, dye containing effluents are considered as one of the most harmful wastewater sources, coming from various industries such as textile, cosmetics, paper, leather, pharmaceuticals or food [2]. Contamination by coloured substances is widely spread, possessing high biochemical and chemical oxygen demand (COD), inadequate pH, turbidity, and toxic chemicals, making the direct effluent discharge to natural reservoirs without further treatment a global problem. If significant savings of potable water are to be achieved in the future, reuse of treated wastewater is one of the main strategies to be employed for this purpose. As a result, development of materials and methods for wastewater treatment that are efficient, cost-effective, and reliable is of paramount importance.

There already exist numerous techniques that allow effective wastewater treatment [3]. However, the most traditional flocculation or coagulation treatment and separation process [4] is still widely used both as a main treatment or as a pre-treatment technique, still making use of an extensive variety of synthetic materials. It is thus critical that this process is optimised to be used effectively in an environmentally-friendly way. However, due to the low biodegradability of the synthetic materials traditionally used in this separation process, which can be detrimental to the environment and/or to health, there is a strong need for their replacement by more eco-friendly alternatives. In particular polymeric flocculants based on natural materials such as lignocellulosic materials have a significant part to play in developing more environmentally-friendly flocculants. Furthermore, using wood wastes as a source for production of such chemical flocculants also allows combining recycling and valorisation of waste materials. However, when using wood-based materials to produce flocculation agents, the main drawbacks to overcome are still their limited chemical reactivity and low water solubility [5].

Among various methods for cellulose functionalization, through the introduction of charged groups into the cellulose backbone, anionization is still insufficiently developed and infrequently used, compared to cationization. The properties of cellulose-based polymers depend on the type of substituent groups, on the degree of substitution (DS) and on their distribution in the cellulose backbone, all of which strongly influence the polymer solubility. Consequently, these features also influence the flocculation ability of the resulting PELs and thus their performance in wastewater treatment.

One of the most known cellulose-based water soluble anionic polyelectrolyte is sodium carboxymethylcellulose (CMC-Na) [6]. The maximum (theoretical) degree of substitution (DS) for CMC-Na is 3.0, but it has been shown that a DS above 0.6 is sufficient to provide good water solubility, and DS values in the range between 0.4 and 1.5 are available in commercial CMC-Na products [7]. However, the application of CMC-Na as flocculant in industrial applications is limited, due to its poor solubility in acidic conditions [8]. A different way to confer anionic character to cellulose polymers is through the introduction of sulfonate groups, in an attempt to match the production of synthetic flocculation agents traditionally used for wastewater treatment. An efficient strategy is to start with a selective oxidation of the cellulose backbone, using, as a first step, a reaction with periodate to increase the reactivity of cellulose and to facilitate the subsequent anionization procedure. Indeed, introduction of reactive aldehyde groups (through the periodate oxidation with production of dialdehyde cellulose) into the cellulose backbone increases the reactivity of cellulose. The pre-modification to dialdehyde cellulose is widely used due to its versatility and efficiency [9,10]. Two aldehyde groups per anhydroglucose (AGU) unit can be introduced to the cellulose backbone using this method, which allows highly modified end products (dialdehyde cellulose) to be obtained [11]. Subsequently, one approach to introduce the anionic charged groups in the cellulose backbone is based on the bisulfite/metabisulfite addition to dialdehyde cellulose (DAC) that allows for the creation of sulfonates. Using this method, Liimatainen et al. [12] produced anionic derivatives of dialdehyde cellulose (ADAC) in a two–step reaction, where bleached birch pulp underwent periodate oxidation, followed by sulfonation with sodium metabisulfite at room temperature, during 24–72 h. The ADACs exhibited 2.1–3.2 mmol/g of charged groups and showed broad pH stability (from pH 3 to 9). This procedure resulted in the production of an effective flocculant for kaolin suspensions. Hou et al. [13] also modified bleached softwood kraft pulp by oxidation/sulfonation, using sodium bisulfite. In another study, by Rajalaxmi et al. [14], DAC obtained from periodate oxidation of hardwood kraft pulp was then also further modified by reaction with sodium bisulfite. It was reported that the solubility of ADAC strongly depends on the sulfonic acid group content, which is driven by the aldehyde group content of the dialdehyde cellulose. In general, the products obtained by using this two–step method demonstrated good water solubility.

A more recent approach was based on the simultaneous combination of periodate and TEMPO (2,2,6,6-tetramethylpiperidine-1-oxyl) in a “one-shot” reaction, to produce 2,3,6-carboxylated cellulose samples via oxidation of hydroxyl groups [15,16]. With this procedure it was also possible to obtain water-soluble fractions of the product but the yield of this fraction was typically low.

The above sequence of periodate oxidation/sulfonation, as far as we know, has never been performed starting from highly complex cellulosic materials, with a high lignin content (high kappa number) to obtain the soluble ADACs.

Thus, the aim of the current study is to establish a procedure to successfully prepare anionic cellulose-based flocculation agents, with different charge (and substitution degree), starting from more complex cellulosic raw materials (with high content of lignin-different kappa numbers), in order to valorise lignocellulosic wastes. A sequence of two reactions: oxidation followed by anionization, following previous studies described in the literature, was adopted. For that, cellulose-rich materials (bleached *Eucalyptus* pulp or pulps from *Eucalyptus* wood wastes obtained by different processes) underwent the oxidation pre-treatment with sodium periodate followed by sulfonation with sodium metabisulfite, at room temperature, keeping the molar ratio of the reactants constant, but studying the influence of reaction time on the final product characteristics. A set of anionic cellulose-based polyelectrolytes was obtained which were then fully characterized. The end-products, anionic natural-based PELs, obtained from each starting material, were evaluated as flocculants in the treatment of both model coloured effluents and a real industrial effluent from the textile industry.

## 2. Materials and Methods

*Eucalyptus globulus* industrial bleached kraft pulp (with lignin content below 0.1 wt%, 85 wt% of cellulose, 14 wt% of glucuronoxylan (hemicellulose) and approximately 1 wt% of other components as ashes or extractives) supplied by The Navigator Company (Cacia, Portugal), was used as the reference sample (hereafter mentioned as bleached cellulosic pulp or C_p_).

*Eucalyptus globulus* industrial wood chips wastes (supplied by The Navigator Company, Cacia, Portugal), with diverse size dimensions, were used as a lignocellulose-rich raw material.

**Figure 1:**
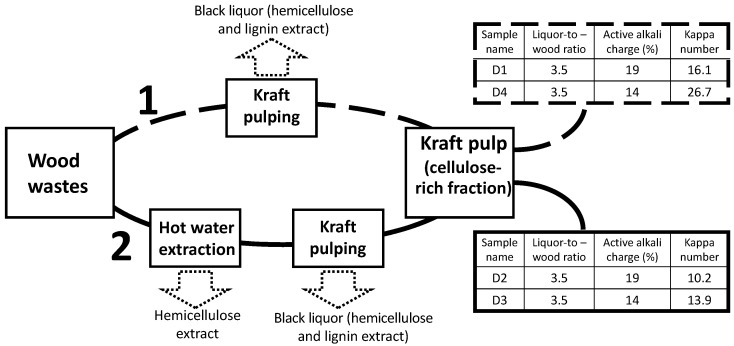
Scheme of *Eucalyptus* wood wastes treatment and resultant pulps (D1–D4).

As described in Figure 1, the *Eucalyptus globulus* wood wastes were treated using two different routes to provide pulps for further modification aiming for diverse chemical complexity of the final PELs. The first route in Figure 1 follows a single step of delignification (lignin removal) by kraft cooking (following which pulps D1 and D4 with different kappa numbers were obtained) while the second route involved a two–step procedure: hot water extraction (partial removal of hemicelluloses and water-soluble constituents) followed by kraft cooking (pulps D2 and D3 were obtained differing in kappa number). Both processes are described in detail below.

### 2.1. Hot Water Extraction of Eucalyptus Wood Wastes

The hot water extraction of Eucalyptus wood chips wastes was performed as previously reported [17]. Briefly, water was added to the wood wastes in a water/wood ratio of 4:1, and the extraction was carried out at 160 °C during 30 min. After cooling, the hot water extracted chips were washed with water and air-dried. This process allowed for the extraction of mainly hemicelluloses and water-soluble constituents.

### 2.2. Kraft Cooking

The kraft cooking of wood wastes was undertaken using a liquor-to-wood ratio of 3.5, at 160 °C for 60 min. The liquor consisted of an aqueous solution of sodium carbonate, sodium hydroxide and sodium sulfide, as reported elsewhere [17]. Two different alkaline charges, of 19 and 14%, were used in the cooking process to provide distinct degrees of delignification (kappa numbers). At the end of the cooking process, the obtained pulps were washed with water and then dried. The same kraft cooking procedures were also applied to the wood chips previously extracted with hot water. Kappa number of the final pulps was measured according to TAPPI Standard T236 om-99. The kraft pulps were analysed for sugar and lignin content. Klason and acid-soluble lignin were determined using the TAPPI Standards T 222 om and T UM 250, respectively, while the sugar content was determined in the hydrolysates using high-performance liquid chromatography (Knauer instrument, Berlin, Germany, with a Smartline pump 1000, Smartline RI Detector S2300, and an Agilent Hi-Plex Ca, 300 mm × 7.7 mm column from Agilent Technologies). It was expected that the two–step procedure (including the hot water extraction, see Figure 1) would lead to products of lower chemical complexity and higher cellulose content as compared to those obtained from the one-step procedure. This is confirmed by the results in Table 1.

Table 1 summarises the chemical composition of the resulting pulps. As expected, different chemical complexity (including different lignin content) in the lignocellulosic materials was obtained by the use of distinct procedures and different concentrations of cooking liquor. Indeed, a higher concentration of cooking liquor (aqueous solution of Na_2_CO_3_, NaOH and Na_2_S) led to products with lower lignin content (Table 1, D2 compared to D3 and D1 compared to D4). Furthermore, the hot water extraction followed by kraft cooking yielded pulps with a significantly lower hemicellulose (xylan) content, and a higher cellulose content (Table 1). Additionally, the purity (based on cellulose content) of samples D2 and D3 obtained here from the two–step process, with pre-hot water extraction, is higher than that of the commercially available Eucalyptus bleached pulp (C_p_), likely as a result of the pre-treatment with hot water.

In principle, a higher lignin content is translated into a higher kappa number. However, when comparing the D1 and D3 pulps, it is worth noting that the D3 pulp, although with a higher lignin content, shows a lower kappa number. In fact, for hardwood pulps, besides lignin, hexenuronic acid groups formed from xylan during the kraft cooking [19] also contribute to the kappa number [20]. The D3 pulp, which has been obtained after a previous hot water extraction of the initial wood sample, presents a substantially lower xylan content (Table 1), and thus less hexenuronic acid moieties. The balance of both contributions (lignin and hexenuronic acids) leads to a slightly lower kappa number for the D3 pulp in comparison to the D1 pulp, in spite of its higher lignin content.

### 2.3. Modification of Pulps with Diverse Lignin Content

#### 2.3.1. Preparation of Dialdehyde Lignocellulose (DAC) by Periodate Oxidation of Pulp

The periodate oxidation followed the procedure described in the literature [21], where 400 g of an aqueous pulp suspension with a consistency of 1%, reacts, during 3 h at 75°C, with 7.2 g of LiCl and 8.2 g of NaIO_4_. The dialdehyde lignocellulose product (DAC) is then filtered and washed several times with distilled water. The as obtained DACs (i.e., not fully dried) from different types of pulps (Table 1) were stored in the fridge and subsequently used for further modification and aldehyde content determination. The aldehyde content of the obtained DACs was determined based on the oxime reaction between aldehyde groups and NH_2_OH∙HCl, as described in the literature [21]. Moreover, oven-dried DAC samples were used for FTIR-ATR measurements.

#### 2.3.2. Preparation of Water-Soluble Anionic Lignocellulose by Sulfonation of DAC

Non-dried DAC (1.5 g on a dry basis) was mixed with 60 mL of distilled water inside a 200 mL round-bottom flask. Then, sodium metabisulfite was added to the mixture in a ratio of 14 mmol bisulfite/g DAC. The reaction mixture was stirred at a controlled temperature of 25 °C with a magnetic stirrer for various times, 24, 34, 72 or 144 h, respectively. The flask was closed with a rubber stopper during the reaction to avoid volume loss and any corresponding concentration changes. After the reaction, the resultant transparent solution was mixed with isopropanol to precipitate the soluble product and the mixture was then centrifuged at 4500 rpm for 45 min. The separated solid was then washed four times or more with a water/isopropanol solution (1/9, *v/v*). The anionic DAC (ADAC-anionic dialdehyde lignocellulose) was oven-dried at 60 °C and then stored in a desiccator. In this process, the reaction time was varied from 24 to 144 h, in order to obtain anionic celluloses with diverse properties (degree of anionization/modification, charge and hydrodynamic diameter) and to be able to evaluate the influence of reaction time on the final product characteristics and performance.

The final anionic products were characterized by Fourier transform infrared (FTIR) and ^1^H nuclear magnetic resonance (NMR) spectroscopies, elemental analysis and light scattering for size (hydrodynamic diameter) and ζ-potential measurements. FTIR-ATR spectra were obtained on a Bruker Tensor 27 spectrometer, using 128 scans and a resolution of 4 cm^−1^, in the range of 4000–600 cm^−1^. ^1^H NMR spectra of the anionic lignocellulose samples dissolved in D_2_O (10 mg mL^−1^) were collected in a Bruker Avance III 400 MHz NMR spectrometer using a Bruker standard pulse program. C, H and S were quantified through elemental analyses using an element analyser EA 1108 CHNS-O from Fisons. 2,5-Bis(5-tert-butyl-benzoxazol-2-yl) thiophene was used as standard. The sulfur content was used to determine the degree of anionization of cellulose in the ADAC samples, each value (S%, *w/w*) being the average of at least 3 measurements.

Hydrodynamic diameter and zeta potential of the anionic lignocellulose materials were determined at 25 °C by dynamic light scattering (DLS) and electrophoretic light scattering (ELS), respectively, in a Zetasizer NanoZS, ZEN3600 (Malvern Instruments, Malvern, UK) with backscatter detection for an angle of 173°. For hydrodynamic diameter measurements, a stock solution of each ADAC with 0.1 g/L concentration, was prepared in Milli-Q water, stirred for 1 h, and then sonicated for 2 min in a sonicator bath. After that process, the anionic lignocellulose sample solution was passed through a 0.45 µm syringe filter directly into the glass measuring cell to eliminate any aggregates. Using the automatic measurement mode, with at least 5 repetitions of each measurement, the average hydrodynamic diameter (nm) of the anionic lignocellulose samples in solution (Z-average diameter) and the PDI (polydispersity index) of the hydrodynamic diameter distribution were obtained. Zeta potential measurements were performed using a 1 g/L stock solution of each ADAC, prepared in Milli-Q water. With a syringe, 1 mL of sample for analysis was carefully injected directly into the disposable plastic capillary cell and measurements, with at least 5 repetitions, were conducted at 25 °C.

### 2.4. Evaluation of Performance in Colour Removal 

#### 2.4.1. Dyes and Industrial Effluent Characterization

In the present study, two dyes were selected for the preparation of model coloured effluents: Crystal Violet (Feldkirch Inc, Feldkirch, Austria), and Methylene Blue (Fluka) considering their wide application in several industries. Their characterization, including ultraviolet/visible spectra, and conductivity of their aqueous solutions, can be found in the literature [21]. The zeta potentials of their aqueous solutions (1 wt%), adjusted to at least three different pH values using hydrochloric acid or sodium hydroxide aqueous solutions, were also measured using ELS in a Zetasizer NanoZS, ZEN3600 (Malvern Instruments, Malvern, UK).

The coloured industrial effluent supplied by Rosarios4 (Mira de Aire, Portugal) was collected after several dyeing sessions, at the end of the dyeing process, containing a mixture of different shades and dyes. The industrial effluent was characterized in terms of COD, pH, turbidity (photometer MD600, Lovibond Tintometer, Amesbury, UK) and zeta potential, the latter being measured after adjustment for different pH values.

#### 2.4.2. Coagulation-Flocculation Experiments

The flocculation performance of the water-soluble anionic lignocelluloses with diverse lignin contents, obtained after 24 h and 72 h of sulfonation reaction, was evaluated using jar-tests. For the flocculation test, a 0.04 wt% ADAC solution was prepared in distilled water. To ensure full dissolution the solution was stirred at 500 rpm for 1 h. The model effluent was prepared by adding the amount of dye to saturate (each dye had a different colour saturation) distilled water followed by stirring at 500 rpm for 30 min. In the experiments, 150 mL of the dye solution was placed in a beaker, and, if required, the pH was adjusted to the target value by adding small volumes of NaOH 10% or HCl 10%. A suitable dosage of flocculant was then added dropwise, while mixing slowly for 20 seconds after flocculant addition. For most of the flocculation experiments, bentonite (with ζ-potential −43 ± 1 mV; size distribution characteristics: d(0.1)–10% undersize percentile diameter of the particle size distribution, 1.5 μm; d(0.5)–median diameter of the particle size distribution, 2.3 μm; d(0.9)–90% undersize percentile diameter of the particle size distribution, 3.4 μm) was also added before the ADAC addition. The jar-test procedure developed for the model effluents was also applied for the industrial effluent using selected anionic natural-based polyelectrolytes and a synthetic flocculant reference, testing different pH values and optimizing the ADAC/bentonite dosage.

For each test and after each dose of flocculant was added, supernatant samples of approximately 10 mL were pipetted from the centre of the jar for determination of colour removal over time (at 1 min, 30 min, 1 h, and 24 h, respectively). The decolouration was evaluated by measuring the turbidity of the supernatant samples, assessed using a photometer MD600 (Lovibond Tintometer, Amesbury, UK) with at least three repetitions. Colour removal was calculated according to Equation (1):

(1) $$Colour\;removal\;(\%)\;=\;\frac{T_{0}-\;T_{f}}{T_{0}}\times 100$$

where *T_0_* is the turbidity of the initial effluent and *T_f_* is the turbidity of the tested sample supernatant at a certain time.

Comparison of the performance of the ADACs produced from the more complex lignocellulosic materials was made with an anionic cellulose-based polyelectrolyte obtained from *Eucalyptus* bleached pulp (ADAC_p_) and with a commercial anionic polyacrylamide (aPAM).

## 3. Results and Discussion

### 3.1. Synthesis and Characterization of Dialdehyde Lignocelluloses and Anionic Lignocelluloses

**Figure 2:**
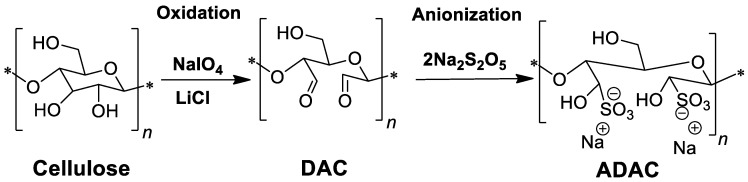
Two-step reaction procedure used to produce anionic lignocellulose-based polyelectrolytes (ADAC).

Modification of *Eucalyptus*-based lignocellulosic materials with diverse lignin contents (see Figure 1) followed a two–step procedure (see Figure 2) as previously described. Following the pulp oxidation with sodium periodate, dialdehyde lignocellulose (DAC) was obtained, which then underwent a sulfonation reaction with sodium metabisulfite, yielding the anionic derivative (Figure 2). The influence of chemical composition (kappa number, cellulose/lignin content) of the used pulps on the final properties of the products obtained was evaluated. Similar paths of anionization, using this sequence of periodate oxidation-sulfonation, have already been described in the literature for different raw materials such as bleached *Betula verrucosa* pulp [12], unspecified commercial bleached hardwood kraft pulp [14] or bleached softwood kraft pulp (*Pinus radiata*) [13]. However, the use of lignocellulosic pulps with high chemical complexity (in terms of lignin content) has not yet been reported in the literature. Nevertheless, the characteristics and properties of the initial cellulose source such as chemical composition and molecular weight and size of the polysaccharide molecules, are important factors that may influence the characteristics of the final anionic products, and thus their performance in targeted applications. In addition, with the increase in lignin content together with the decrease of uniformity in the distribution of the molecular size of the initial polysaccharides, chemical modifications may be more difficult and adjustments may be required. Additionally, evaluation of longer sulfonation times up to 144 h, as reported in this work, and its influence on diverse features of the obtained anionic PELs is also of importance to have a better picture of the influence of reaction conditions on the final product characteristics, and has not been described previously in the literature.

**Figure 3:**
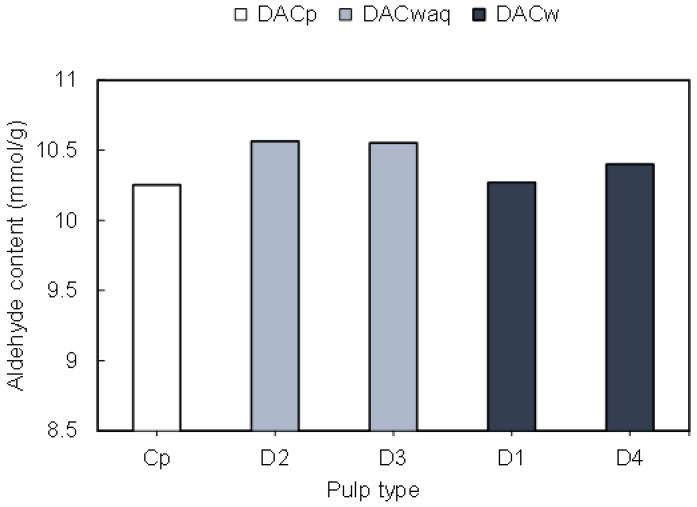
Influence of different lignin content of the raw materials on the aldehyde group content after the periodate oxidation. The reaction conditions were kept constant in all experiments at 75 °C and 3 h. DACp corresponds to dialdehyde cellulose obtained from bleached pulp, DACwaq to dialdehyde lignocellulose obtained from pulp prepared by hot water extraction/kraft cooking, and DACw corresponds to dialdehyde lignocellulose obtained from pulp submitted only to kraft cooking.

Water solubility of the obtained products could be achieved through a high functionalization degree (3–5 mmol/g), when introducing a high content of negatively charged sulfonate groups in the cellulosic backbone. Moreover, introduction of two highly reactive aldehyde groups per AGU unit during the periodate oxidation step proved to be crucial in the production of the lignocellulose-based PELs through the described route. In general, the results of periodate oxidation performed on pulps with diverse heterogeneity (*Eucalyptus* bleached pulp and kraft pulps with or without hot water pre-treatment with high kappa numbers) did not demonstrate a significant impact of the initial pulp characteristics on the obtained oxidised products, when applying optimised modification conditions (75 °C, 3 h) with periodate excess (9.6 (mmol) NaIO_4_/(g) pulp ratio) (Figure 3, results with a maximum deviation of 3%, minimum three replica). Analysing the results of Figure 3 in more detail, the DAC_p_ obtained from *Eucalyptus* bleached pulp (C_p_) possessed ca. 10.25 mmol of aldehyde groups /g (determined based on the nitrogen content of the oxime derivative produced by reaction with NH_2_OH∙HCl, as explained in the experimental section). When applying the same oxidation procedure for a set of more heterogeneous lignocellulosic pulps obtained by kraft cooking of wood wastes with hot water pre-treatment (series of DAC_waq_ samples obtained from initial pulps D2 and D3) or without hot water pre-treatment (DAC_w_ samples obtained from initial pulps D1 and D4), similar degrees of oxidation were reached (between 10.2 and 10.6 mmol of aldehyde groups/g). Thus, results of periodate oxidation show that using more complex raw materials, with eventually lower cellulose content and with higher content of other constituents such as lignin, had a limited impact on the obtained oxidised products, for the Kappa numbers considered.

The highly oxidised dialdehyde cellulose derivatives obtained (DACs) were further anionized in order to produce anionic bio-PELs (ADACs). This reaction leading to the introduction of negatively charged sulfonate groups occurred between the aldehyde functionalities and sodium metabisulfite as described in Figure 2. The obtained products were characterized and further studied as flocculants in both model and industrial effluents. The sulfonation reaction time was varied from 24 h to 144 h, while keeping all the other parameters constant such as the molar ratio of sodium metabisulfite to aldehyde and the reaction temperature (room temperature −25°C). It is likely that, if the aldehyde content of DAC is higher and a longer reaction time is used, a higher extent of derivatisation of aldehyde groups in DAC to sulfonate groups and a higher anionic content will be obtained, as described below. The variation of reaction times (24–144 h) was anticipated to lead to natural-based polyelectrolytes with different degrees of anionization, and indeed anionicity indices ranging from 3.61 to 4.90 mmol/g were obtained (Table 2).

**Figure 4:**
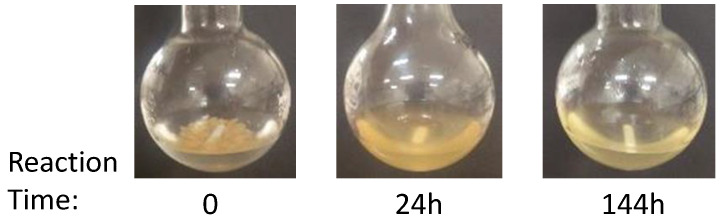
Visual observation of the mixture in the sulfonation reaction of DAC_waq_D3 before starting the reaction (time 0), and after 24 h (ADAC_D3_A) and 144 h (ADAC_D3_D) of reaction time.

It should be noted, based on the visual inspection shown in Figure 4 that the appearance of products obtained, while using the same metabisulfite/aldehyde ratio, differed with the reaction time. Indeed, as reaction time increased, a greater homogeneity in the product was observed. Solubility was confirmed by the total transparency of the reaction solution at the end of the modification procedure. With an increase in kappa number of the raw materials, a 24 h reaction time appeared to be insufficient for a complete solubilisation of the modified pulp. In fact, for the ADACs _D3_A, _D1_A and _D4_A, it was noticed that the reaction mixture was still cloudy after 24 h, and the obtained products were not fully soluble and thus the mixture required further filtration to recover the water-soluble fraction only. It can be concluded, that when lignin is present, penetration of sodium metabisulfite in the dialdehyde lignocellulose sample is more limited and longer reaction times are required to provide sufficient sulfonation (anionization) in the final product.

The first main conclusion is that, under all the conditions tested, a high degree of sulfonate group substitution was achievable thus allowing PELs with high anionicity index to be synthesised. However, according to the analytical determinations of sulfur content in the ADACs, higher reaction times were not necessarily translated into higher sulfonation degrees. Furthermore, results of the sample characterization for anionic DACs derived from the same DAC, e.g., DAC_p_ (ADAC_p_: A, B, C, D), suggested that for reaction times longer than 72 h the product undergoes chemical degradation, which leads to a decrease of the resulting polyelectrolyte hydrodynamic diameter. Also, it is important to bear in mind that cellulose and other chemical constituents, such as hemicelluloses, can undergo a reaction with sodium metabisulfite in their dialdehyde form. Overall, 34–72 h of sulfonation reaction time proved to be optimum for the best compromise between the degree of substitution and size of the chain (related to the hydrodynamic diameter value). This is the case for ADACs obtained from pulps with higher cellulose content (ADAC_p_, ADAC_D2_, ADAC_D3_) and also for ADAC_D4_ (see Table 1), leading to cellulose derivatives with the most promising characteristics (high sulfonation degree, large hydrodynamic diameter and highly negative zeta potential) for further application as flocculation agents. For all the ADACs, except for ADAC_D1_, the lower hydrodynamic diameters recorded when higher reaction times (144 h) are used, suggests that degradation of the polymer chains occurs. The polydispersity index of the anionic PELs hydrodynamic diameter distribution was between 0.28 ± 0.01 and 0.59 ± 0.08. Such high PDI values confirm the large heterogeneity (size and thus molecular weight) of the products obtained after anionization.

Bearing in mind the importance of a high content of ionic groups to increase water solubility of the PELs, as well as the economic feasibility of the reaction procedure (reaction time as the main parameter) from a commercial perspective, the cellulose derivatives produced after 24 h and 72 h, obtained from each tested pulp, were selected to be further evaluated as potential natural-based flocculation agents for dye removal in both model systems and the coloured industrial effluent. 

**Figure 5:**
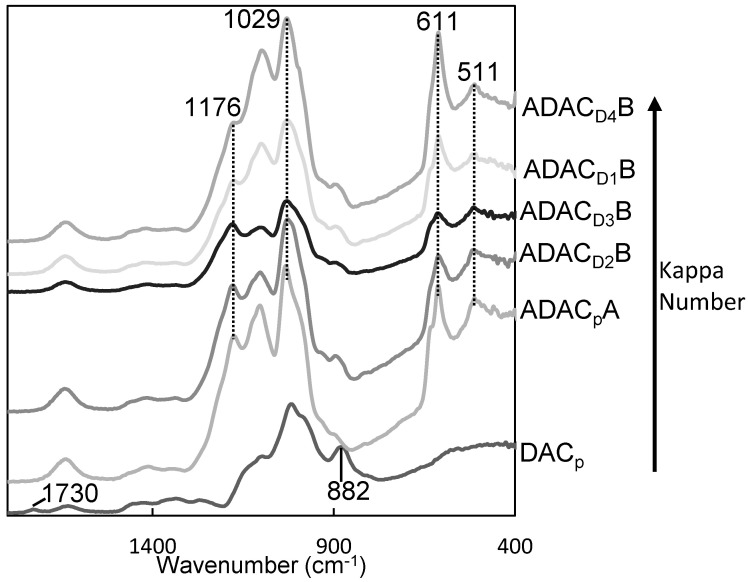
FTIR spectra of dialdehyde cellulose (DAC_p_) and anionic lignocelluloses obtained from different sources of dialdehyde lignocellulose after 34 h of sulfonation (ADAC_p_A, ADAC_D2_B, ADAC_D3_B, ADAC_D1_B and ADAC_D4_B).

Figure 5 presents an example of a FTIR spectrum of a DAC intermediate product, where a new band, at ca. 1730 cm^−1^ corresponding to the C = O stretching of aldehyde groups is evident. This band was not observed in the spectra of the initial lignocellulosic pulps. This confirmed the success of the periodate oxidation reaction, including the ring opening and oxidation of OH groups at the C2−C3 positions of the AGU unit, as previously reported and in agreement with other works [11,12,17,22].

**Figure A1:**
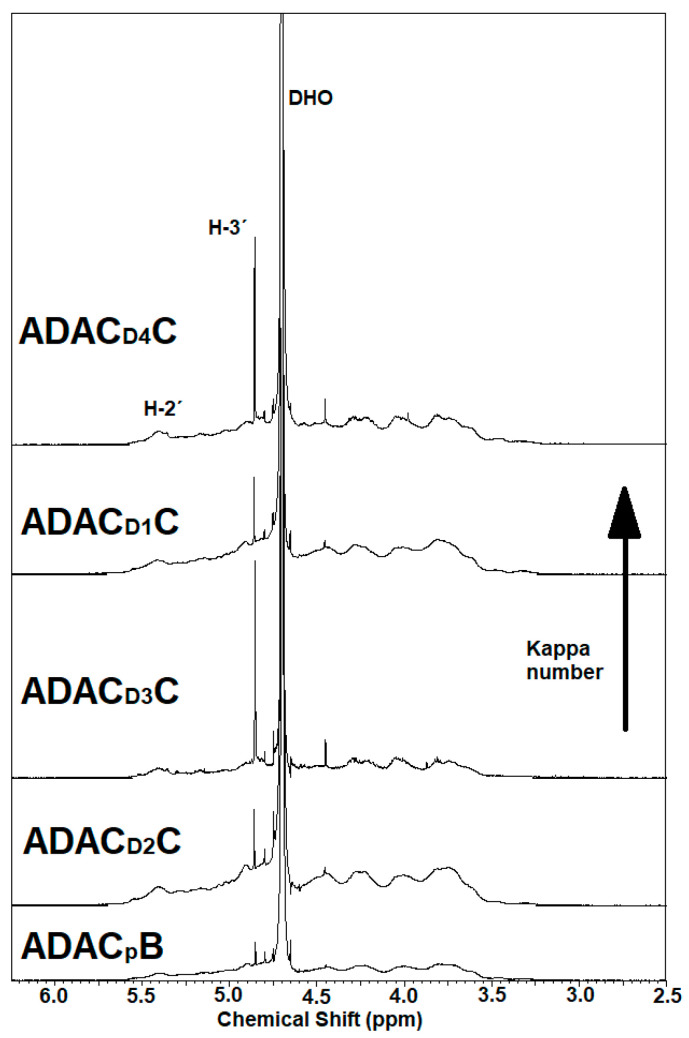
^1^H NMR spectra of anionic lignocellulose-based derivatives obtained from different sources of dialdehyde lignocellulose after 72 h of sulfonation (ADAC_p_B, ADAC_D2_C, ADAC_D3_C, ADAC_D1_C and ADAC_D4_C).

After the anionization through sulfonation of DACs, the final products were characterized by both FTIR and ^1^H NMR spectroscopy as presented in Figure 5 and Figure A1 (Appendix A). FTIR spectroscopy proved to be a useful technique to confirm the structural modification of DAC samples by sulfonation. Figure 5 shows the FTIR spectra of the anionic lignocellulosic derivatives. Appearance of several new bands at 1183–1175 cm^−1^, 615–609 cm^−1^ and 517–506 cm^−1^ was associated with SO_2_ and S−O vibrations of sulfonic acid groups (asymmetric S(=O)_2_ stretching, S−O stretching and possibly S(=O)_2_ wagging, respectively). The highest intensity band of DAC at 1015 cm^−1^ was also shifted to 1036–1027 cm^−1^, as the result of the introduction of sulfonate groups in the polysaccharides structure (symmetric S(=O)_2_ stretching). Moreover, the disappearance of the DAC characteristic band at 1730 cm^−1^ confirmed the success of the sulfonation reaction.

The ^1^H NMR spectra of the water-soluble sulfonated lignocellulose derivatives produced from DACs obtained from different initial pulp samples (samples ADAC_p_B, ADAC_D2_C, ADAC_D3_C, ADAC_D1_C and ADAC_D4_C) are presented in Figure A1. The ^1^H NMR spectra are not very illustrative since the obtained signals were broad and highly overlapped with the signal from water. However, based on the characterization performed by Rajalaxmi et al. [14] it was possible to distinguish two main signals assigned to H-2′ (bonded to C2) and H-3’ (bonded to C3) directly connected to the SO_3_^−^ groups. Moreover, due to the oxidation/sulfonation of C2−C3, the signals were shifted, and appeared at 5.4 and 4.85 ppm, respectively.

Therefore, ^1^H NMR and FTIR spectroscopy gave clear evidence that anionization of cellulosic materials with diverse chemical composition, through the sulfonation reaction of DACs with sodium metabisulfite, occurred.

Zeta potential of water-soluble, anionic cellulosic PELs was between −32 mV and −50 mV, which confirmed the success of the derivatisation process and the production of negatively charged polyelectrolytes. However, even if the anionicity index (3.61–4.90 mmol/g) differed significantly between ADACs obtained from initial wood pulps with kappa number between 10.2 and 26.7, this did not show a very high influence in the zeta potential of the polymers that varied between −36 ± 2 mV and −46 ± 1 mV. Moreover, in the series ADAC_D4_, obtained from the initial material with the highest kappa number (26.7), the measured zeta potential results were also in the range of −46 ± 1 mV to −36 ± 2 mV being similar to the zeta potential values of ADAC_D2_, obtained from the initial material with the lowest kappa number (10.2), which were between −41 ± 2 mV and −36 ± 1 mV. This suggests a low impact of lignin on the modification process. Only in the case of ADAC_p_, obtained from bleached *Eucalyptus* fibres, composed mainly of cellulose and hemicellulose, with insignificant lignin content, the zeta potential of the anionic polyelectrolytes obtained was slightly higher (maximum −50 mV).

Moreover, it was also noticeable a tendency relating hydrodynamic diameter and reaction time, in materials obtained from initial bleached pulp as well as from wood pulp submitted to hot water extraction and the lowest kappa number (10.2), where with the increase of sulfonation time the hydrodynamic diameter decreases, most possible, as the result of chains degradation. This possibility is also aligned with the decrease of PDI (polydispersity index) of the ADACs obtained from these two pulps, when reaction time increases. For the bio-PELs obtained from more heterogeneous raw materials this trend is not so notorious, as referred previously, probably due to the role lignin plays during the modification process, which can limit the cellulose chain degradation. Furthermore, all the produced anionic modified DACs obtained from bleached pulp and pulps with different lignin contents showed to be water soluble at room temperature, and thus can be strong candidates to be applied as natural-based flocculants in wastewater treatment.

### 3.2. Decolouration Studies (Dye Removal)

#### 3.2.1. Model Effluents

The results of the decolouration tests using the developed anionic PELs, obtained after 24 h and 72 h of sulfonation reaction, will be presented here for the two tested model effluents and discussed individually for each dye system.

**Figure 6:**
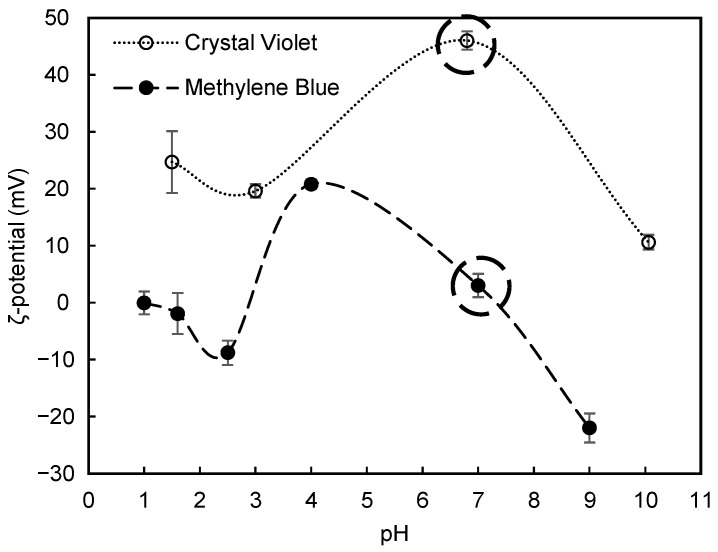
Zeta potential for the two model effluents tested as a function of pH. The initial zeta potential of the dye without pH adjustment (at around neutral pH) is highlighted. The lines are drawn to guide the eye of the reader.

The large availability of different types of dyes and shades leads to large amounts of wastewaters which are extremely challenging to treat, due to the unique chemical structures of the dyes, with positively or negatively charged organic chromophores and relatively small molecular weight. In addition, it is important to bear in mind that changes in pH can lead to ionization of certain groups and therefore to a variation of the charge of the dye, depending on the pH range. The zeta potential of the tested dyes as a function of pH was determined experimentally and is shown in Figure 6, where the initial value for each dye is specifically highlighted. Both the zeta potential of Methylene Blue as well as that of Crystal Violet showed to be pH dependent. Different performances for the tested flocculants are then expected to be observed with the change of pH, when treating the model wastewaters containing these dyes.

- Methylene Blue

**Figure 7:**
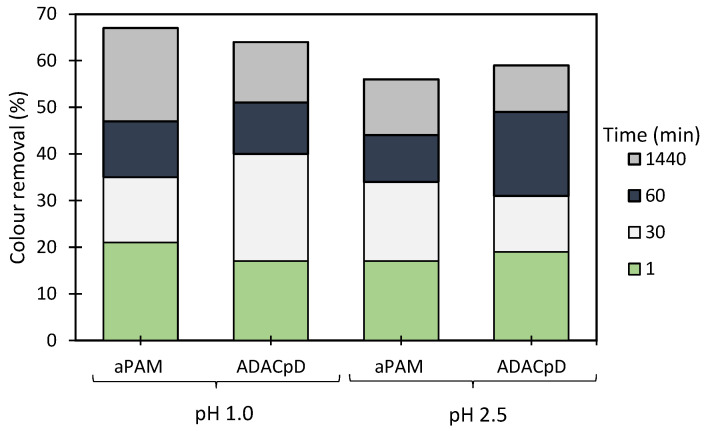
Methylene Blue colour removal using the flocculation agent alone (2.67 mg/L of synthetic aPAM or cellulose-based ADAC_p_D) at two different pH levels, 1.0 and 2.5.

**Figure 8:**
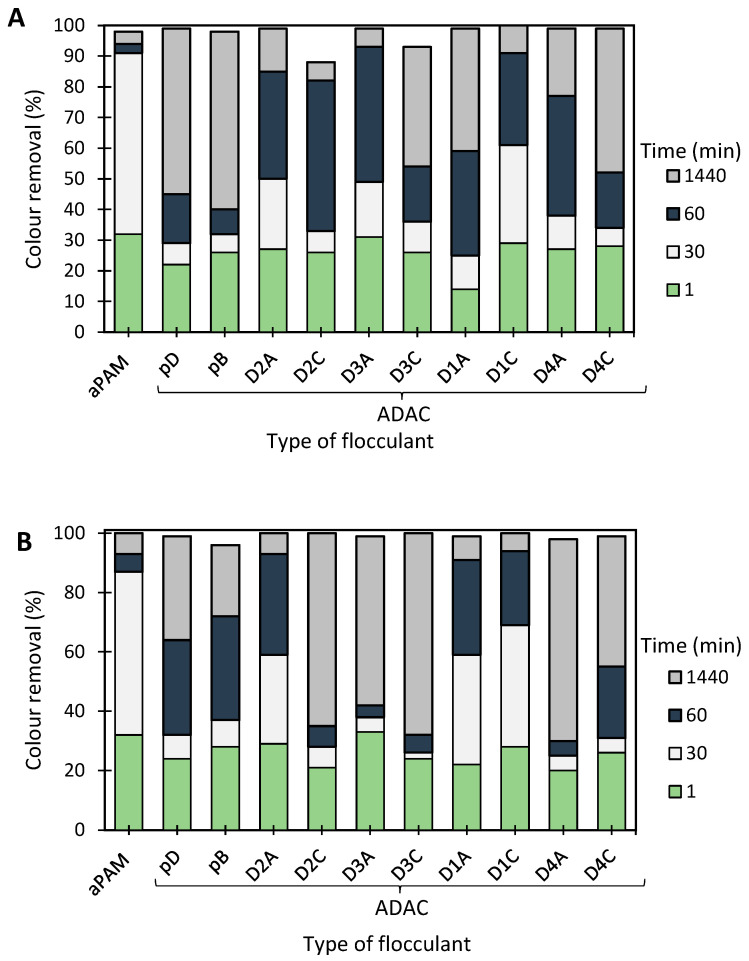

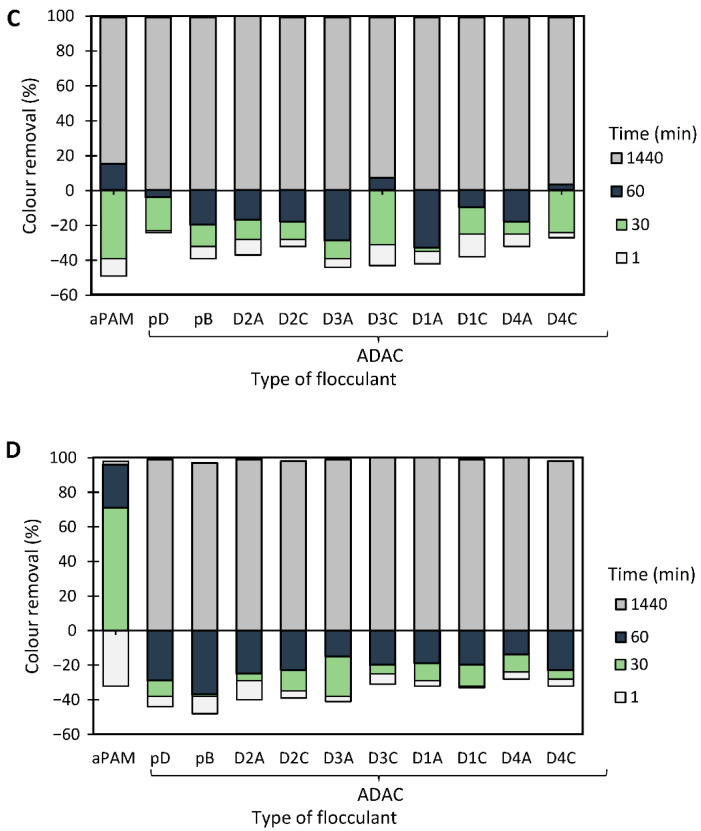
Methylene Blue colour removal for different treatment times, using cellulose-based flocculation agents from different sources in dual system with bentonite at pH 1.6. Procedure (**A**): 0.07 wt% of bentonite followed by 1.33 mg/L of flocculant. Procedure (**B**): 0.07 wt% of bentonite and 2.67 mg/L of flocculant. Procedure (**C**): 0.14 wt% of bentonite followed by 1.33 mg/L of flocculant. Procedure (**D**): 0.14 wt% of bentonite and 2.67 mg/L of flocculant. Anionic synthetic PAM (aPAM) in dual system with bentonite was used as reference. ADACs are identified according to Table 2.

**Figure 9:**
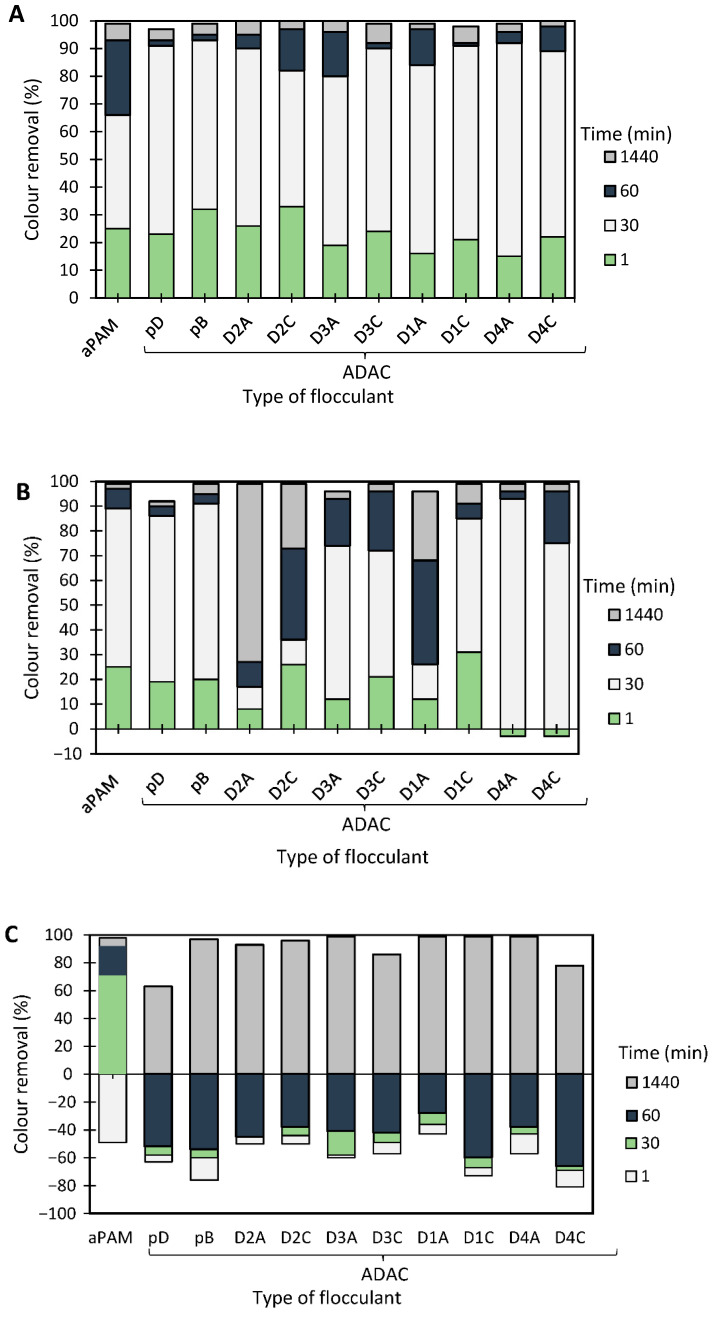

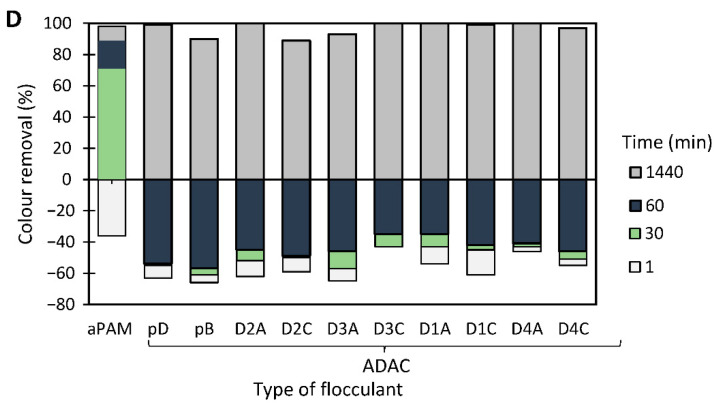
Methylene Blue colour removal for different treatment times, using cellulose-based flocculation agents from different sources in dual system with bentonite at pH 2.5. Procedure (**A**): 0.07 wt% of bentonite followed by 1.33 mg/L of flocculant. Procedure (**B**): 0.07 wt% of bentonite and 2.67 mg/L of flocculant. Procedure (**C**): 0.14 wt% of bentonite followed by 1.33 mg/L of flocculant. Procedure (**D**): 0.14 wt% of bentonite and 2.67 mg/L of flocculant. Anionic synthetic PAM (aPAM) in dual system with bentonite was used as reference. ADACs are identified according to Table 2.

The results of the Methylene Blue model effluent treatment, based on the turbidity reduction, obtained while using the anionic flocculation agents developed from *Eucalyptus* raw materials of different cellulose, lignin, and hemicellulose contents, are summarized in Figure 7, Figure 8 and Figure 9. The initial colour removal trials were carried out using single systems, with only ADAC_p_D (anionic cellulose-based PEL obtained from bleached pulp after 24 h of sulfonation reaction) or aPAM (synthetic anionic PEL of similar charge) (Figure 7). In addition, a range of different pHs was used to evaluate the influence of pH on the colour removal. In these preliminary tests, acidic pHs were considered (pH 1.0 and 2.5), due to poor results (no colour removal) when working at neutral or alkaline pH levels, because of the higher stability of the tested model dye-water system for those pHs. The decolouration results at pH 1.0 or at pH 2.5 were good overall and no difference could be noted between the cellulose-based and synthetic flocculation agents. Moreover, either aPAM or ADAC_p_D were able to remove above 60% of the dye at pH 1.0 and above 55% of the dye at pH 2.5 after 24 h of treatment. Nevertheless, to try to increase colour removal, dual systems with bentonite were tested.

**Figure 10:**
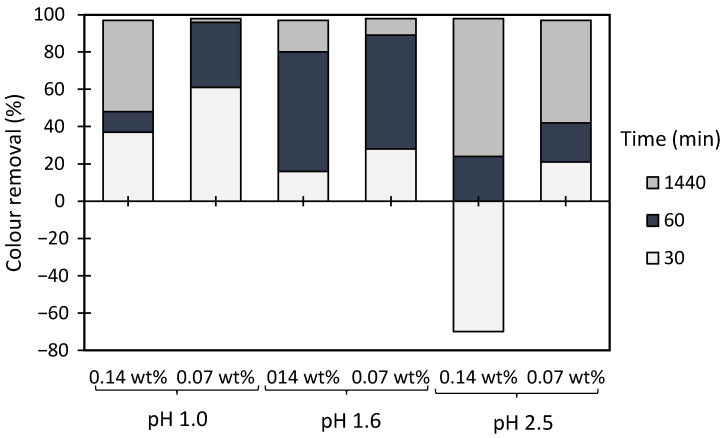
Methylene Blue colour removal with time, using 0.07 wt% or 0.14 wt% of bentonite alone at different pH values, 1.0, 1.6 and 2.5.

Figure 8 and Figure 9 show the results obtained at pH 1.6 and 2.5, while using two different concentrations of flocculation agent: 1.33 mg/L (procedures A and C) or 2.67 mg/L (procedures B and D), and two different amounts of bentonite: 0.07 wt% (procedures A and B) or 0.14 wt% (procedures C and D). Additionally, procedures A–D were compared with the treatment performed only with bentonite in the absence of flocculation agent (see Figure 10). Comparison with the reference synthetic flocculant, aPAM, in dual system with bentonite, is also included in all the Figure 8 and Figure 9.

In all the presented tests, colour removal increased with time, thus largest colour removal was obtained at the end of the 24 h test. Although Methylene Blue is typically positively charged, for lower pH values (pH < 3) the dye is more unstable possessing a lower zeta potential (see Figure 6) and this was the reason for the selection of such low pH levels to perform this study. It was observed that at pH 2.5, while applying a dual system with bentonite followed by the anionic cellulose-based flocculants, almost full colour removal (≥99%) was obtained in most of the experiments, for the longest settling time (24 h).

At the tested pHs (1.0 to 2.5) bentonite alone allowed good colour removal (see Figure 10), after 24 h, even if for pH 2.5 (the most interesting one) removal could be slightly lower when using bentonite alone rather than the dual system. Moreover, for this pH, and under the best conditions (0.07 wt% of bentonite), settling was slower when using bentonite alone rather than the dual system (compare Figure 9 A,B with Figure 10). Additionally, increasing the amount of bentonite, when used alone, was detrimental to colour removal kinetics (Figure 10).

Analysing in more detail the results obtained for the dual systems (Figure 8 and Figure 9), similar good decolouration results could be obtained for pH 1.6 and 2.5, not only for the longest settling time (at 24 h of treatment), but also for intermediate settling times (compare Figure 8A,B and Figure 9A,B). However, flocculation was faster (higher removal after 30 min of treatment) when working at pH 2.5, using procedures A or B (0.07 wt% of bentonite followed by 1.33 mg/l of flocculant or 0.07 wt% of bentonite followed by 2.67 mg/l of flocculant, respectively), in particular in the case of procedure A. The influence of pH was more noticeable when a higher amount of bentonite was used, for which slightly lower removals were obtained at higher pH (compare Figure 8C,D and Figure 9C,D for pH 1.6 and 2.5, respectively). Additionally, increasing the amount of bentonite from 0.07 wt% to 0.14 wt%, did not improve decolouration. The procedure with 0.07 wt% of bentonite followed by 1.33 mg/L of ADAC provided good decolouration results, with no significant benefit appearing also from increasing the polymers concentration from 1.33 to 2.67 mg/l (compare A and B in Figure 8 or in Figure 9). When working at higher bentonite dosages, 0.14 wt% (see Figure 8C,D and Figure 9C,D) in dual system, for shorter contact times (up to 1 h) synthetic aPAM appeared to be more effective. However, after 24 h of settling both the synthetic and the cellulose-based flocculation agents showed very similar flocculation efficiency. 

When applying the dual system, primarily, bentonite adsorbs the dye, and the turbidity of the system can even increase at the very beginning of the process, due to the presence of non-settled bentonite, leading to negative values of colour/turbidity removal, whereas later addition of the polymer triggers flocculation, based on the bridging mechanism, and leads to rapid settling. In all the tested procedures, for different pH levels, the underlying mechanism appeared to be the same. 

Typically, in this coloured effluent, decolouration efficiency obtained with anionic cellulose-based flocculation agents showed to be as good as for the synthetic aPAM reference. When comparing the performance of the different ADACs tested, there is no clear pattern relating their characteristics, especially the purity of the initial fibres used for the modification, to flocculation performance. Indeed, the ADACs obtained from the pulp with the highest kappa number (lignin content) such as ADAC_D4_A or ADAC_D4_C were as effective in the decolouration of Methylene Blue, as the other evaluated cellulose-based flocculation agents obtained from higher purity cellulosic pulps. One can thus conclude that anionic lignocellulose-based flocculants work well for this purpose, independently of the raw material used in the modification procedure.

- Crystal Violet

**Figure 11:**
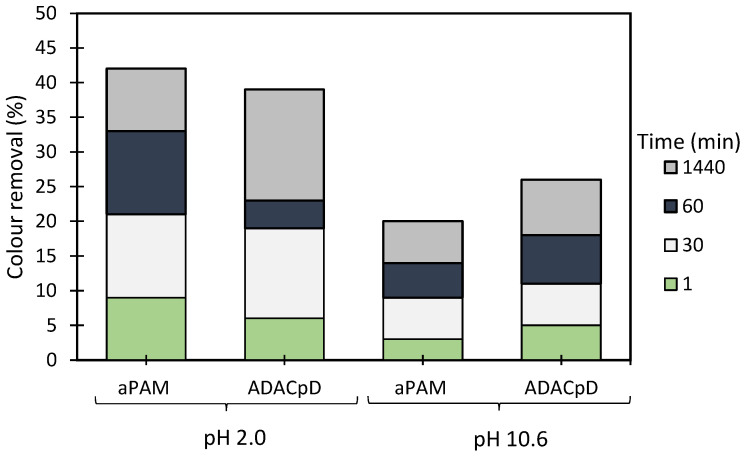
Crystal Violet colour removal using a single system with only flocculation agent (2.67 mg/L of synthetic aPAM or cellulose-based ADAC_p_D) at two different pH levels, 2.0 and 10.6.

**Figure 12:**
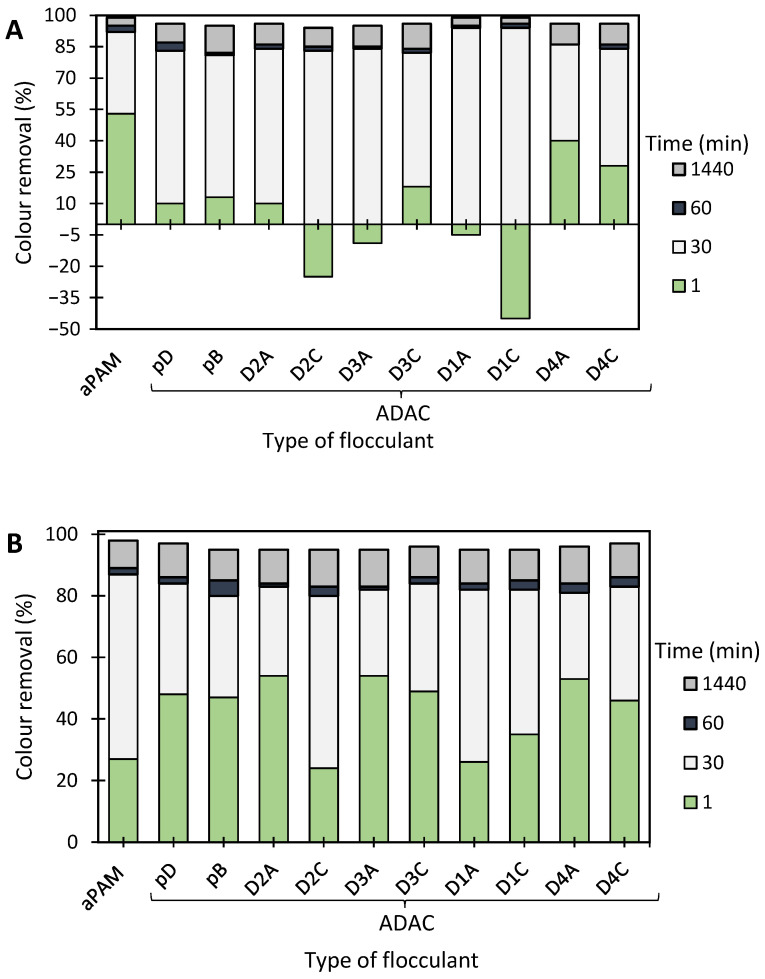
Crystal Violet colour removal for different times, using cellulose-based flocculation agents from different sources in dual system with bentonite at pH 2.0. Procedure (**A**): 0.07 wt% of bentonite followed by 1.33 mg/L of flocculant. Procedure (**B**): 0.14 wt% of bentonite and 1.33 mg/L of flocculant. Anionic synthetic aPAM in dual system with bentonite was used as reference. ADACs are identified according to Table 2.

**Figure A2:**
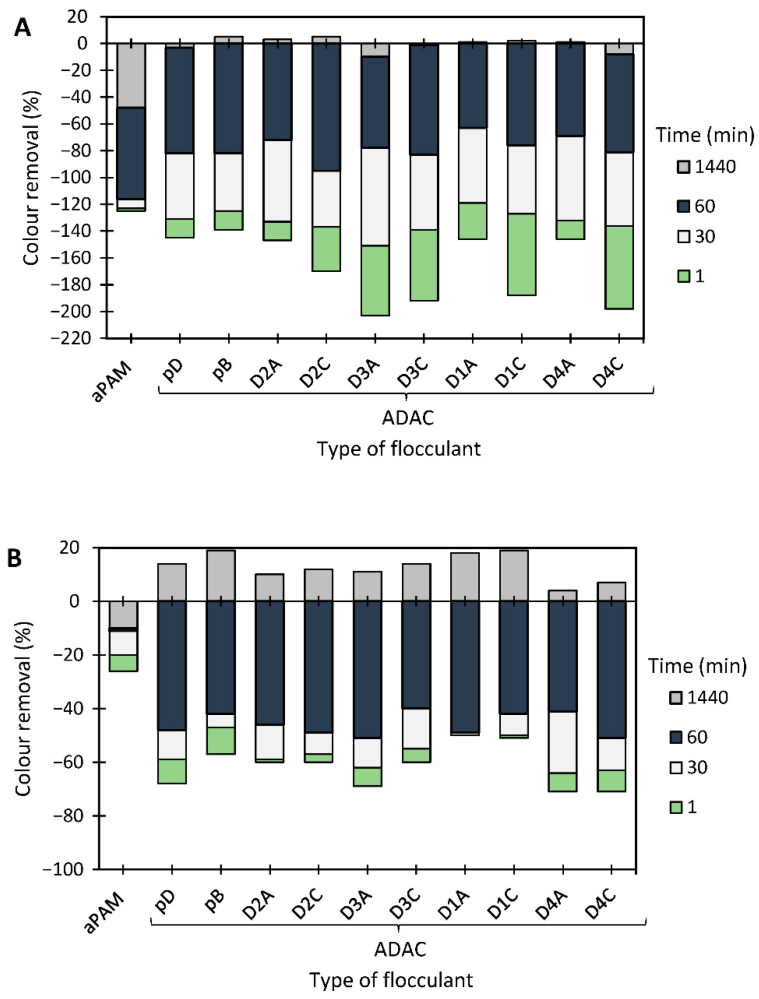
Crystal Violet colour removal for different times, using cellulose-based flocculation agents from different sources in dual system with bentonite at pH 6.0. Procedure (**A**): 0.07 wt% of bentonite followed by 1.33 mg/L of flocculant. Procedure (**B**): 0.14 wt% of bentonite and 1.33 mg/L of flocculant. Anionic synthetic aPAM in dual system with bentonite was used as reference. ADACs are identified according to Table 2.

**Figure A3:**
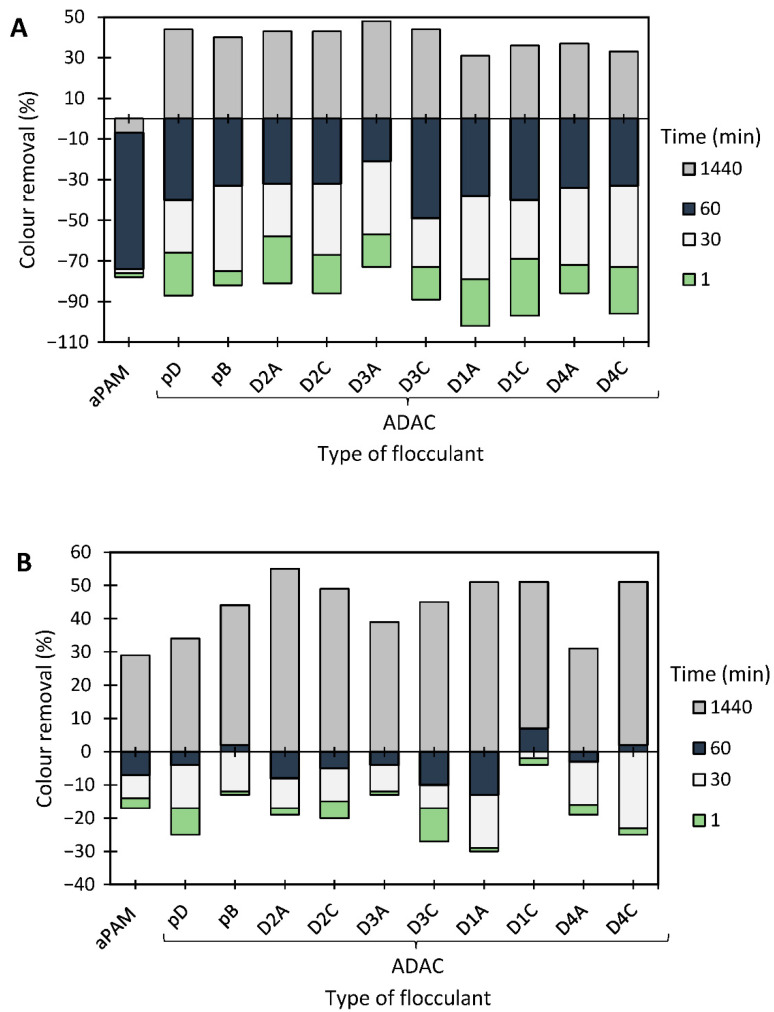
Crystal Violet colour removal for different times, using cellulose-based flocculation agents from different sources in dual system with bentonite at pH 10.6. Procedure (**A**): 0.07 wt% of bentonite followed by 1.33 mg/L of flocculant. Procedure (**B**): 0.14 wt% of bentonite and 1.33 mg/L of flocculant. Anionic synthetic aPAM in dual system with bentonite was used as reference. ADACs are identified according to Table 2.

The results obtained for the Crystal Violet model effluent using anionic cellulose-based flocculation agents produced from *Eucalyptus* raw materials of different cellulose and lignin contents, are summarized in Figure 11 and Figure 12, Figure A2 and Figure A3. Results of preliminary tests of Crystal Violet decolouration, using the polymer alone (anionic cellulose-based PEL obtained from bleached pulp after 24 h of sulfonation reaction (ADAC_p_D) or aPAM (synthetic PEL of similar charge)) are presented in Figure 11. Different pH values were used to perform trials using this model effluent so as to evaluate the influence of pH on the flocculation ability and efficiency of the tested polymers. Crystal Violet is positively charged (see Figure 6) but varying pH can promote a charge variation of the dye. It was then important to evaluate the performance of the produced polyelectrolytes at an acidic pH (2.0) and at alkaline pH (10.6), considering the different stability of the system in these conditions.

Figure 11 shows Crystal Violet decolouration results at two different pHs (2.0 and 10.6) using 2.67 mg/L of ADAC_p_D or synthetic aPAM, without addition of any complexing agent. Decolouration was better at pH 2.0 for both cellulose-based and synthetic flocculation agents. Moreover, aPAM was marginally more effective in the treatment of this dye for acidic conditions, removing 42% of colour, while ADAC_p_D removed only 39%, after 24 h of treatment. However, for the alkaline conditions, better colour removal was observed with ADAC_p_D (26% after 24 h) comparing to aPAM (20% after 24 h). Nevertheless, the polymer alone was not enough to achieve an adequate colour removal. Thus, once again, dual systems with bentonite were studied in order to intensify the decolouration ability of the tested flocculants.

**Figure 13:**
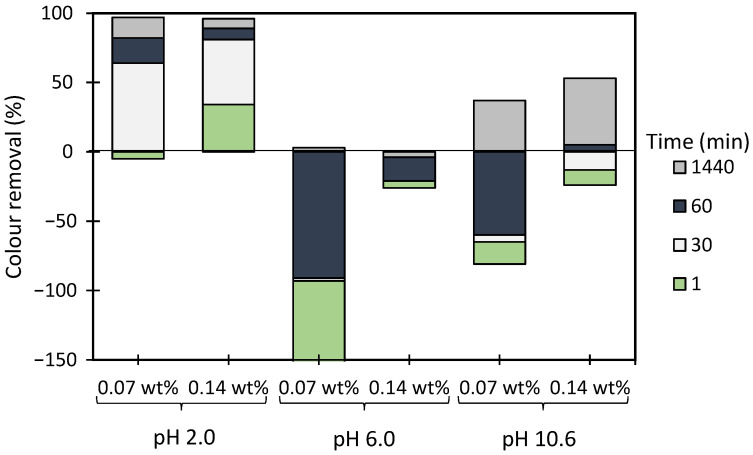
Crystal Violet colour removal for different times, using 0.07 wt% or 0.14 wt% of bentonite alone at different pH values: 2.0, 6.0 and 10.6.

Figure 12, Figure A2 and Figure A3 show the results obtained at pH 2.0, 6.0 and 10.6, while using two different amounts of bentonite: 0.07 wt% (procedure A) or 0.14 wt% (procedure B), followed by the addition of 1.33 mg/L of the flocculation agent. Additionally, procedures A and B were compared with the treatment performed only with bentonite for the same amounts of this component (see Figure 13). In all the tests, colour removal increased with time, with the best results obtained at the end of the 24 h tests. Crystal Violet is positively charged for all pHs examined herein, however, at pH 6.0 (near the initial pH of the model system) the system showed to be the most stable (see Figure 6). At this particular pH, the tests showed the worst decolouration results (Figure A2), with null or very low colour removal (maximum 20%) being achieved. At the lowest pH, while applying dual systems, colour removal reached the highest values (removals between 91 and 100% after 24 h). Typically, as referred to previously, the addition of bentonite destabilises the dye-water system and addition of an anionic polymer allows bridging between bentonite-dye complexes, therefore flocs become larger, and faster settling over time occurs. Additionally, in the dual system flocculation with the ADACs at pH 2.0 (Figure 12), colour removal was already high (from 80 to 90%) after 30 min of treatment (procedures A, B), characteristic of an efficient flocculation. Furthermore, at this pH, increasing the amount of bentonite (compare A and B in Figure 12) did not lead to a significant increase in colour removal, except in the first minute of treatment. Performance of the ADACs was similar to that of the reference synthetic aPAM.

At pH 10.6, bentonite presents a negative surface charge [23], which was not completely neutralized by the positively charged dye molecules in this tested system. This resulted in less effective decolouration while using ADACs (anionic PEL) combined with bentonite, when compared with colour removals at pH 2.0, in particular at lower settling times. The reference aPAM presented worse decolouration results for higher pH values when compared to the tested anionic natural-based flocculants. 

For all the situations tested (pH and amount of bentonite used), the dual system always performed better than when bentonite alone was used (compare Figure 12, Figure A2 and Figure A3 with Figure 13).

In summary, after the bentonite complexation with the dye, turbidity of the system usually increases, but, as some sites on the bentonite surface remain without being complexed with the dye, they are available for further polymer adsorption, followed by bridging, and effective flocculation occurs thereafter. The most effective Crystal Violet removal was obtained while working at pH 2.0 with a lower amount of bentonite (0.07 wt%), followed by 1.33 mg/L of polymer (ADAC_D1_A, ADAC_D1_C or aPAM), which allows for a colour removal near 100% after 24 h of treatment and faster flocculation kinetics (higher removal for 30 min of treatment). Generally, by increasing the effluent acidity, the system tends to be more unstable and flocculation treatment with the ADACs in these conditions leads to higher decolouration levels. In addition, as in the case of the Methylene Blue removal tests, a clear trend relating the characteristics of the initial raw material (pulp) used to produce the ADACs and their performance in flocculation was not observed. It is worth stressing that the lignocellulose-based PELs obtained from the pulp with the highest kappa number (ADAC_D4_A and ADAC_D4_C) were as effective as the synthetic reference or other ADACs obtained from pulps with lower chemical complexity, when working at pH 2.0. It is also worth noting that, at pH 10.6, the ADACs proved to be more effective than the synthetic reference. Moreover, the success of colour removal showed to be highly pH dependent, due to ionization of functional groups in the chemical structure of the dye, as well as due to the influence the pH can have on the inorganic additive, bentonite, used in the process. 

In general, colour removal for the two model effluents tested was very high and similar in acidic conditions, using the polymer (ADACs) with a concentration of 1.33 mg/l and a dosage of bentonite of 0.07 wt%, even if flocculation was slightly faster (after 1 min of treatment) in the case of methylene blue, which agrees with the higher instability of this dye at acidic pH (see Figure 6).

#### 3.2.2. Industrial Effluent

The flocculation performance of the developed anionic polyelectrolytes in multi-coloured industrial effluent treatment was evaluated using PELs obtained after 24 and 72 h of sulfonation reaction. This industrial effluent (COD 0.687 gO_2_/L, pH 4.5 and turbidity 57 NTU) was collected from several dyeing/washing sessions, at the end of the dyeing process, thus containing a mixture of different shades and dyes, being representative of a real wastewater from the textile industry. 

**Figure 14:**
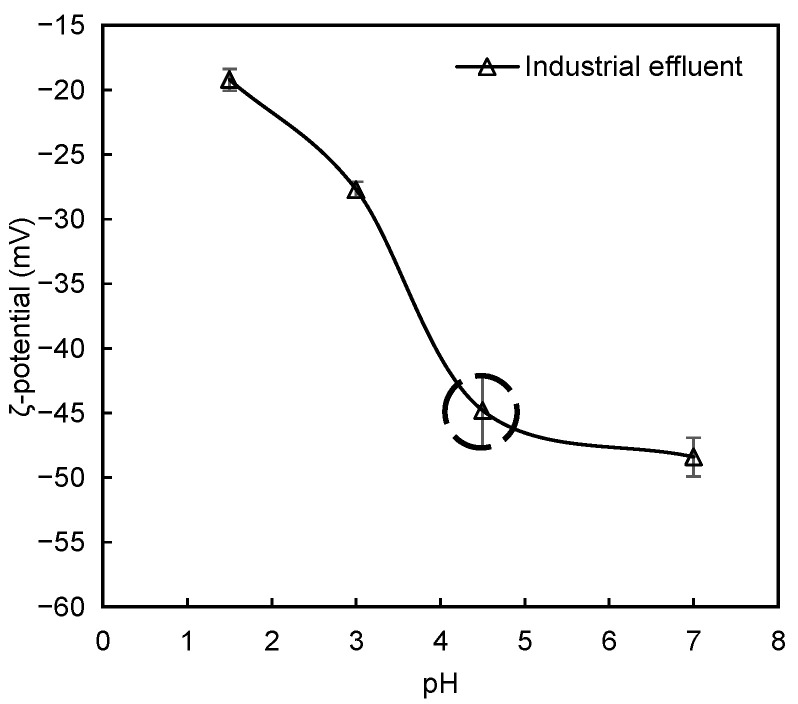
Zeta potential for the industrial effluent as a function of pH. The initial zeta potential without pH adjustment is highlighted. Line is drawn to guide the eye of the reader.

The stability of dye-containing effluents is determined mainly by particle charge, which is affected by the pH of the medium as was seen in the model systems described previously. The zeta potential of the multi-colour effluent after pH adjustments (initial value is highlighted) was measured, and the obtained values with accompanying standard deviations are presented in Figure 14. In this industrial effluent, the particles are mainly negatively charged. With the increase of pH, a significant increase in negative charge of the particles was observed. The highly negatively charged dye molecules and other suspended solids of this system promote stabilization of the effluent, making it more difficult to destabilize and treat at alkaline pH levels. At neutral pH and at the initial pH 4.5, the zeta potentials were sufficiently high (−48 mV and −45 mV, respectively) to likely provide strong repulsive interactions between the particles. It is then expected that better colour removals can be obtained for acidic conditions where the effluent possesses a lower charge, thus being more unstable.

**Figure 15:**
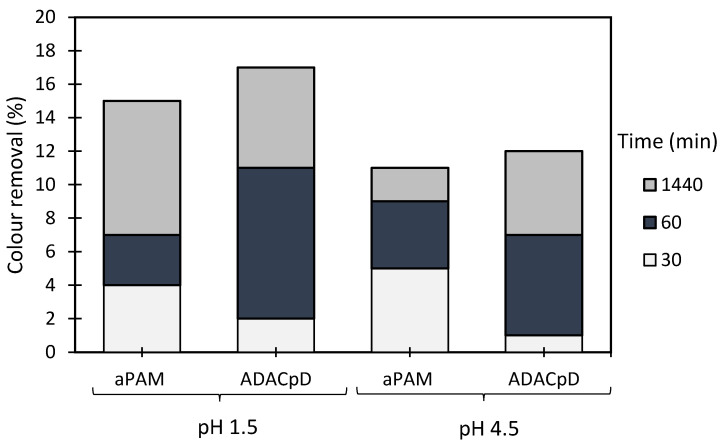
Industrial coloured effluent treatment using a single system only with flocculation agent (5.34 mg/L of synthetic aPAM or cellulose-based ADAC_p_D) at two different pH levels, 1.5 and 4.5 (initial).

**Figure 16:**
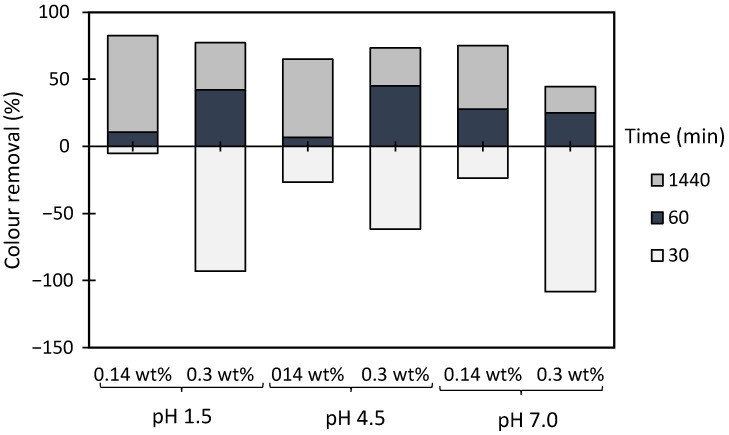
Industrial effluent treatment for different times, using 0.14 wt% or 0.3 wt% of bentonite alone at different pH values: 1.5, 4.5 (initial) and 7.0.

**Figure 17:**
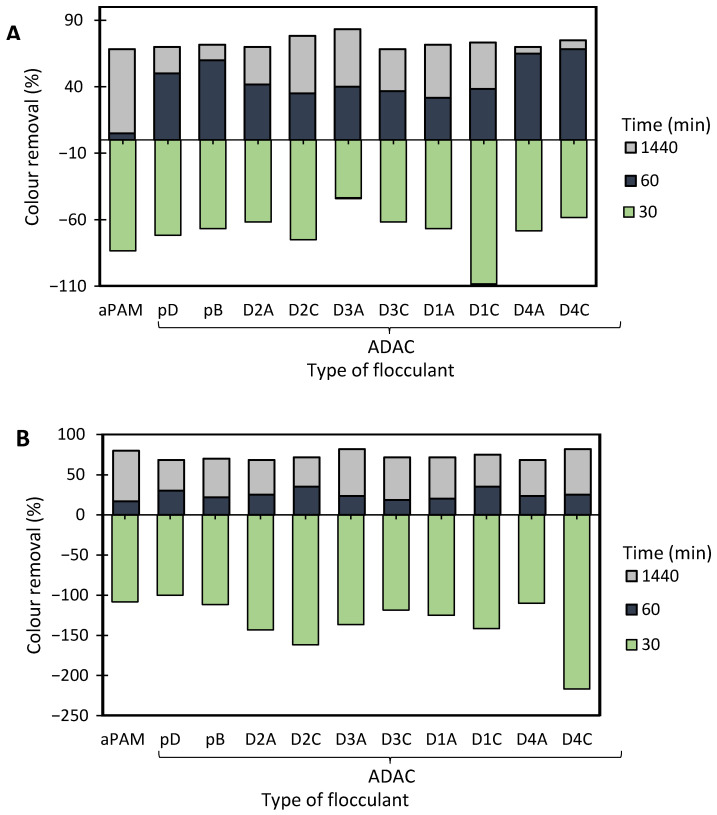
Industrial effluent treatment for different times, using cellulose-based flocculation agents from different sources in dual system with bentonite at initial pH 4.5. Procedure (**A**): 0.3 wt% of bentonite followed by 2.67 mg/L of flocculant. Procedure (**B**): 0.3 wt% of bentonite and 5.34 mg/L of flocculant. Anionic synthetic aPAM in dual system with bentonite was used as reference. ADACs are identified according to Table 2.

**Figure 18:**
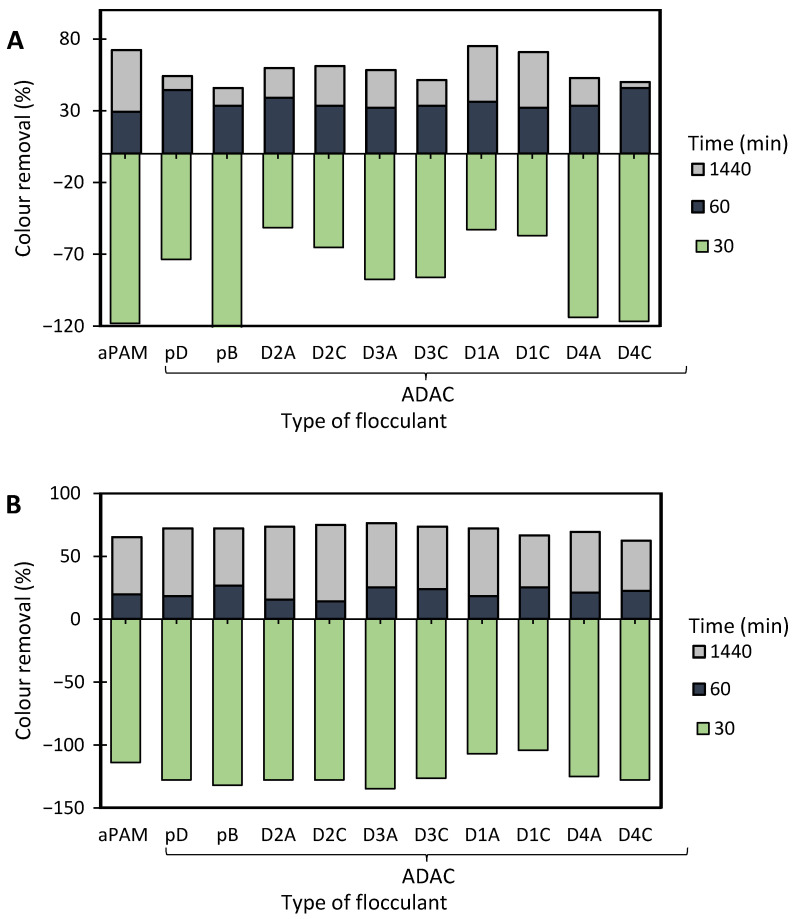
Industrial effluent treatment for different times, using cellulose-based flocculation agents from different sources in dual system with bentonite at pH 7.0. Procedure (**A**): 0.3 wt% of bentonite followed by 2.67 mg/L of flocculant. Procedure (**B**): 0.3 wt% of bentonite and 5.34 mg/L of flocculant. Anionic synthetic aPAM in dual system with bentonite was used as reference. ADACs are identified according to Table 2.

The results of the industrial effluent treatment, based on the turbidity reduction, obtained while using the developed anionic flocculation agents from *Eucalyptus* raw materials of different cellulose and lignin contents, and aPAM (synthetic anionic PEL of similar charge), are summarized in Figure 15, Figure 16, Figure 17 and Figure 18. In this case the supernatant water turbidity over time for 30 min, 1 h and 24 h, was used to evaluate the PELs performance in the treatment of this industrial effluent. Due to the negative charge of the particles of the effluent at different pHs, poor flocculation was expected while using only the negatively charged PELs. This was confirmed while performing tests, using single systems only. Indeed, using the anionic natural-based (ADAC_p_D) or synthetic (aPAM) polyelectrolytes on their own at a concentration of 5.34 mg/L and at two different pHs, namely pH 1.5 and pH 4.5 (initial effluent pH) led to poor colour removal (see Figure 15**).** In this case, the highest colour removal was obtained with ADAC_p_D at pH 1.5 (17% turbidity reduction after 24 h). At pH 1.5, the effluent tends to be less stable (zeta potential of −19 mV), as compared to the initial pH 4.5 (zeta potential of −45 mV), which minimizes the repulsion effect between particles. Although, some turbidity reduction was obtained while using these single systems, the clarification results were poor and the removals were never higher than 20% after 24 h of treatment. Thus, dual systems combining the anionic PELs with the previously used inorganic complexation agent (bentonite) were also tested.

In general, applying the dual system (bentonite-flocculant) (Figure 17 and Figure 18) increased flocculation efficiency, when compared to results obtained using a single system, either bentonite alone (Figure 16) or the flocculant alone (Figure 15), for the same pH and dosages. For all tested pH levels (initial 4.5, 1.5 and 7.0) bentonite alone was not effective enough to clarify the effluent (see Figure 16). Moreover, when using bentonite on its own, at the initial pH of the effluent (pH 4.5), a higher turbidity reduction was achieved with 0.3 wt% of bentonite (close to 70%) and thus this dosage was also selected when performing the tests with the dual systems. In addition, since the initial pH (4.5) and neutral pH led to comparable results when using bentonite alone, it was decided to use these two pHs in the tests with the dual systems. In these tests (Figure 17 and Figure 18), bentonite starts by destabilizing the system interacting with the negatively charged particles/dyes suspended in the effluent, due to bentonite slightly positively charged surface, leading, thereafter, to higher turbidity in the effluent because of the presence of the unflocculated bentonite, which justifies the negative colour removal values during the first period of treatment (up to 30 min). Further addition of polymer allows for flocs formed of bentonite particles and adsorbed dyes to grow, increasing the settling rate, and thus colour removal rate also increases. Furthermore, using dual systems with natural-based anionic flocculants led to similar clarification results as compared to those with the synthetic anionic reference (aPAM), for the same conditions. Moreover, working at initial pH 4.5 allowed better clarification results (colour removal after 24 h reaching 85% when using procedure A), as compared to results obtained at pH 7.0 (colour removal reaching 75% after 24 h for the same conditions). Generally, procedure B did not appear to lead to higher colour removal levels than procedure A (higher polymer dosage in procedure B) for both pHs. This means that for this effluent and for the conditions tested, increasing the polymer dosage does not favour colour removal which may be due to the increase of the steric effect which hinders flocculation.

Additionally, pH influences the selection of the most effective anionic cellulose-based PEL, as it appears to be different according to the pH conditions (particularly with procedure A). However, the flocculants obtained from *Eucalyptus*-pulp with the highest kappa numbers (higher lignin content) and the larger hydrodynamic diameters (ADACs D4, ADACs D1 and ADACs D3), typically showed the best performance among tested PELs for the different conditions tested. This was clearly noticeable for pH 7.0 in particular for ADACs D1 in conditions A. Since flocculation is more difficult for this pH, a higher hydrodynamic diameter associated with a higher molecular weight may favour flocculation, particularly when the bridging mechanism is relevant. Also, a higher hydrophobic/lignin fraction, still unmodified in the sample, can interact better with the negatively charged effluent impurities. It is also worth stressing that for the initial pH (4.5) the two ADACs D4 (higher lignin content in the raw fibres) led to much faster colour removal in conditions A (see Figure 17).

**Figure 19:**
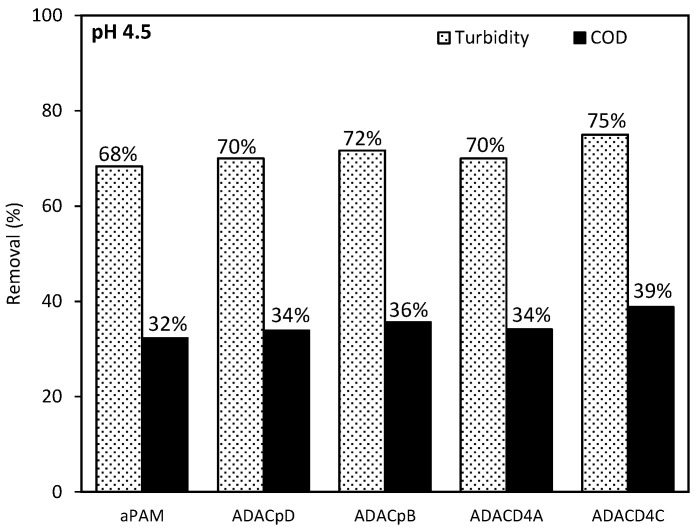
COD reduction and turbidity reduction for treatment of multi-coloured industrial effluent with ADACs (ADAC_p_D, ADAC_p_B, ADAC_D4_A, ADAC_D4_C) and reference aPAM, at pH 4.5, after 24 h of treatment, using 0.3 wt% bentonite followed by 2.67 mg/L of flocculation agent (Procedure A).

Anionic natural-based PELs, produced from bleached pulp, ADAC_p_D, obtained after 24 h of sulfonation, and ADAC_p_B obtained after 72 h of reaction, as well as anionic cellulose-based PELs obtained from pulp with the highest lignin content, ADAC_D4_A (24 h of modification reaction) and ADAC_D4_C (72 h of modification reaction), and the reference polymer aPAM, were tested also for COD reduction after 24 h of treatment. The conditions used for the COD measurements refer to the initial pH of 4.5, 0.3 wt% bentonite and the lower concentration of flocculant, corresponding to procedure A (0.3 wt% bentonite followed by 2.67 mg/L of flocculation agent), since this was the procedure which led to better results (see Figure 19). The COD reduction obtained with the tested new anionic bio-polyelectrolytes (ADACs) was poor, in the range of 34–39% (with turbidity reduction of 70–75% for these polymers and procedure), while with the synthetic reference performance in COD reduction was still lower, 32%. However, it is known that flocculation alone does not usually lead to high COD reductions when treating this type of industrial effluents [24].

Considering the overall results, the negative charge of the tested industrial effluent, seems to have a crucial influence on the performance of the flocculation process using the developed anionic polyelectrolytes, as expected. However, previous studies by the authors [25], showed that for this type of highly coloured effluents containing small dyes’ molecules, the addition of an inorganic complexation agent is always required, even if oppositely charged polymers are used. Thus, based on the results obtained it is possible to state that the anionic cellulosic PELs developed proved to be a good and promising solution for the treatment of this highly complex effluent, especially considering that they work better for lower concentrations and either for the initial effluent pH or even at neutral pH (pH 7.0), as long as they are combined with an intermediate inorganic component of opposite charge (e.g., bentonite).

## 4. Conclusions

This work reported a two–step modification procedure of *Eucalyptus* pulps, differing in chemical composition (hemicellulose, cellulose and lignin contents) to produce, at laboratory scale, water-soluble anionic lignocellulose-based polyelectrolytes (PELs). Periodate oxidation followed by sulfonation with sodium metabisulfite allowed to produce anionic PELs from various types of *Eucalyptus*-based cellulosic raw materials. Also, sulfonation time (while keeping other reaction parameters constant) showed to be a key factor in obtaining products with diverse characteristics. This study revealed that the optimum reaction time was 34–72 h since longer reaction times seemed to promote the chemical degradation of the cellulose chains, in particular when starting from more homogeneous cellulosic raw materials. Negatively charged natural-based PELs with zeta potential in the −32 to −50 mV range were obtained. In general, the anionic lignocellulose-based PELs were also easily solubilized in water at room temperature.

Those PELs were applied as flocculation agents in wastewater treatment (dyes removal). These anionic dialdehyde lignocelluloses (ADACs), showed to have a high potential as flocculation agents. Moreover, a dual system with bentonite, was found to be more efficient for colour removal of the tested model dye effluents: Methylene Blue and Crystal Violet solutions. In fact, the dual system with bentonite followed by the flocculation agent, was generally required to obtain high colour removals, i.e., above 90%. pH proved to have a strong effect on the flocculation process, and working in acidic pH conditions allowed for higher colour removals and rapid flocculation kinetics. The anionic cellulosic polymers developed also proved to be effective for colour and turbidity reduction when treating a highly complex multi-coloured industrial effluent, which was very stable at initial (4.5) or neutral pH (very negative zeta potential). For this purpose, it was also necessary the introduction of an inorganic complexation agent, as an aid, prior to the flocculation process, which significantly increased the destabilisation of the effluent and promoted adsorption of the dye molecules. Overall, the flocculants obtained from the pulp with the highest kappa number (lignin content of 4.4%) provided very good results, similar to the ones obtained when using the bio-PELs (ADACs) obtained from raw materials with higher chemical homogeneity and lower lignin content. Similar or better results were obtained while using the natural-based flocculants when comparing to a reference commercial anionic polyacrylamide, normally used for this purpose, applied under the same test conditions.

In summary, new anionic lignocellulose-based materials can be proposed as a more environmentally friendly alternative for the treatment of dye-containing effluents using the flocculation process. Using the proposed lignocellulose-based flocculants as wastewater treatment agents, can be considered as a very attractive valorisation of natural wastes (namely *Eucalyptus* wood wastes) with significant positive environmental impacts.

## Figures and Tables

**Table 1:** Chemical analysis of the kraft pulps used as raw materials for the synthesis of dialdehyde lignocellulose.

Name	Kappa Number	Cellulose Content (wt%)	Xylan Content (wt%)	Total Lignin Content (wt%)
**Commercially available *Eucalyptus* bleached kraft pulp ^a^**				
C_p_	_	85	14	<0.1
***Eucalyptus*** **wood waste pulps ^b^**				
D2	10.2	90.2	7.3	1.9
D3	13.9	89.6	7.2	2.8
D1	16.1	76.7	17.5	2.2
D4	26.7	71.6	18.3	4.4

C_p_: commercially available bleached pulp; D2 and D3: pulps obtained by kraft cooking with previous hot water extraction of the wood wastes; D1 and D4: pulps obtained by kraft cooking; **^a^** values taken from the literature [18]. **^b^** xylan content does not include substituent acid groups.

**Table 2:** Reaction conditions for synthesis of anionic bio-PELs (ADACs) and characterization results of final products.

Name	Time (h)	Temp (°C)	Anionicity Index (mmol/g)	ζ-Potential (mV)	Z-Av. Diameter (nm)	PDI
**DAC_p_**	3	75				
ADAC_p_D	24	25	4.23	−44 ± 2	157 ± 10	0.59 ± 0.08
ADAC_p_A	34	25	4.44	−44 ± 2	178 ± 2	0.52 ± 0.03
ADAC_p_B	72	25	4.17	−50 ± 1	127 ± 6	0.52 ± 0.02
ADAC_p_C	144	25	4.60	−43 ± 1	92 ± 3	0.32 ± 0.03
**DAC_waq_D2**	3	75				
ADAC_D2_A	24	25	3.66	−41 ± 2	151 ± 4	0.44 ± 0.01
ADAC_D2_B	34	25	4.03	−38 ± 1	155 ± 6	0.43 ± 0.02
ADAC_D2_C	72	25	3.61	−38 ± 1	135 ± 7	0.48 ± 0.02
ADAC_D2_D	144	25	4.04	−36 ± 1	108 ± 8	0.42 ± 0.01
**DAC_waq_D3**	3	75				
ADAC_D3_A	24	25	4.62	−43 ± 2	140 ± 3	0.34 ± 0.04
ADAC_D3_B	34	25	4.07	−42 ± 3	138 ± 3	0.28 ± 0.01
ADAC_D3_C	72	25	4.44	−32 ± 2	175 ± 8	0.30 ± 0.03
ADAC_D3_D	144	25	4.28	−44 ± 1	114 ± 4	0.29 ± 0.03
**DAC_w_D1**	3	75				
ADAC_D1_A	24	25	4.17	−41 ± 4	106 ± 4	0.34 ± 0.05
ADAC_D1_B	34	25	3.96	−42 ± 3	170 ± 7	0.43 ± 0.05
ADAC_D1_C	72	25	4.11	−42 ± 2	163 ± 8	0.41 ± 0.02
ADAC_D1_D	144	25	4.03	−38 ± 3	173 ± 6	0.47 ± 0.03
**DAC_w_D4**	3	75				
ADAC_D4_A	24	25	4.47	−45 ± 1	137 ± 8	0.47 ± 0.02
ADAC_D4_B	34	25	4.72	−46 ± 1	162 ± 8	0.45 ± 0.03
ADAC_D4_C	72	25	4.59	−39 ± 1	157 ± 5	0.37 ± 0.03
ADAC_D4_D	144	25	4.90	−36 ± 2	116 ± 7	0.46 ± 0.03

Anionicity determined as the amount of sulfonate groups (mmol) per g (dry weight) of anionic lignocellulose sample; PDI-polydispersity index of the hydrodynamic diameter distribution; DAC_waq_ refers to DAC obtained from fibres resulting from hot water extraction and kraft cooking; DAC_w_ refers to DAC obtained from fibres resulting only from kraft cooking.

## Data Availability

The data presented in this study are available on request from the corresponding author. The data are not publicly available due to lack of adequate repository.
